# Inhibiting Lysine Demethylase 1A Improves L1CAM-Specific CAR T Cell Therapy by Unleashing Antigen-Independent Killing via the FAS-FASL Axis

**DOI:** 10.3390/cancers13215489

**Published:** 2021-10-31

**Authors:** Ornela Sulejmani, Laura Grunewald, Lena Andersch, Silke Schwiebert, Anika Klaus, Annika Winkler, Kathy Astrahantseff, Angelika Eggert, Anton G. Henssen, Johannes H. Schulte, Kathleen Anders, Annette Künkele

**Affiliations:** 1 Department of Pediatric Oncology and Hematology, Berlin Institute of Health, Charité–Universitätsmedizin Berlin, Corporate Member of Freie Universität Berlin, Humboldt Universiät zu Berlin, 10353 Berlin, Germany; ornela.sulejmani@charite.de (O.S.); laura.grunewald@charite.de (L.G.); lena.andersch@charite.de (L.A.); silke.schwiebert@charite.de (S.S.); anika.klaus@charite.de (A.K.); Annika.winkler@charite.de (A.W.); Kathy.astrahantseff@charite.de (K.A.); angelika.eggert@charite.de (A.E.); anton.henssen@charite.de (A.G.H.); johannes.schulte@charite.de (J.H.S.); 2 German Cancer Consortium (DKTK), 10117 Berlin, Germany; kathleen.anders@charite.de; 3 German Cancer Research Center (DKFZ), 69120 Heidelberg, Germany; 4 Experimental and Clinical Research Center, Lindenberger Weg 80, 13125 Berlin, Germany

**Keywords:** neuroblastoma, pediatric cancer, adoptive immunotherapy, epigenetic regulation, solid tumors, antigen-independent tumor cytotoxicity

## Abstract

**Simple Summary:**

Solid tumor cells can lose or heterogeneously express antigens to become resistant to chimeric antigen receptor (CAR) T cell therapy. Here, we explore whether epigenetic manipulation to unleash antigen-independent killing mechanisms can overcome this hurdle. KDM1A is overexpressed in many cancers and removes lysine methylation on histones that keeps the DNA firmly packed to selectively activate or repress gene activity, depending on the specific lysine target. KDM1A also regulates the expression of nonhistone proteins. We inhibited KDM1A in the childhood tumor, neuroblastoma, to increase FAS expression on tumor cells. The FAS receptor can be triggered to induce cell death when bound by the FAS ligand on CAR and other activated T cells present in the tumor environment, even if the tumor cells lack the target antigen. FAS upregulation via KDM1A inhibition sensitized neuroblastoma cells to FAS-FASL-mediated killing and augmented CAR T cell therapy against antigen-poor or even antigen-negative neuroblastoma.

**Abstract:**

Chimeric antigen receptor (CAR) T cell therapy has emerged as a promising treatment strategy, however, therapeutic success against solid tumors such as neuroblastoma remains modest. Recurrence of antigen-poor tumor variants often ultimately results in treatment failure. Using antigen-independent killing mechanisms such as the FAS receptor (FAS)-FAS ligand (FASL) axis through epigenetic manipulation may be a way to counteract the escape achieved by antigen downregulation. Analysis of public RNA-sequencing data from primary neuroblastomas revealed that a particular epigenetic modifier, the histone lysine demethylase 1A (KDM1A), correlated negatively with FAS expression. KDM1A is known to interact with TP53 to repress TP53-mediated transcriptional activation of genes, including *FAS*. We showed that pharmacologically blocking KDM1A activity in neuroblastoma cells with the small molecule inhibitor, SP-2509, increased FAS cell-surface expression in a strictly TP53-dependent manner. FAS upregulation sensitized neuroblastoma cells to FAS-FASL-dependent killing and augmented L1CAM-directed CAR T cell therapy against antigen-poor or even antigen-negative tumor cells in vitro. The improved therapeutic response was abrogated when the FAS-FASL interaction was abolished with an antagonistic FAS antibody. Our results show that KDM1A inhibition unleashes an antigen-independent killing mechanism via the FAS-FASL axis to make tumor cell variants that partially or totally suppress antigen expression susceptible to CAR T cell therapy.

## 1. Introduction

An innovative immunotherapeutic approach to target tumors is adoptive T cell therapy, which hijacks the immune system to direct effector mechanisms against metastatic and resistant tumor cells. Autologous T cells redirected to a specific tumor-associated antigen by introducing a chimeric antigen receptor (CAR) hold great therapeutic promise. CARs are chimeric molecules that combine an antibody-derived extracellular single-chain antigen-binding domain with intracellular signaling and costimulatory domains from the T cell [1]. The most successful CAR T cell therapy to date received FDA approval in 2017 and targets CD19, a B cell linage-specific antigen whose expression is retained in most B cell malignancies [2]. Despite the unprecedented efficacy of CD19-CAR T cell therapy, a number of patients still experience relapses due to heterogeneous CD19 expression in the tumor cells, leading to outgrowths of therapy-resistant tumor variants that have lost or downregulated CD19 expression [3]. Therapy resistance is even more likely to occur in solid tumors, which show greater heterogeneity in target antigen expression [4,5,6,7]. Major barriers to the success of CAR T cell therapy against neuroblastoma comprise suboptimal T cell persistence, a lack or poor expression of tumor-specific targets, and an immunosuppressive tumor microenvironment, among other factors [8]. New strategies to enhance potency are necessary to improve CAR T cell therapeutic success.

Cytotoxic T cells kill target cells mainly by two major pathways, either by exocytosis of cytotoxic granules that contain perforin and granzymes or by FAS-FASL-mediated induction of apoptosis. CAR T cells mainly kill tumors by the perforin and granzyme pathway [9,10]. However, blocking FASL in malignant T-cell acute lymphoblastic leukemia (T-ALL) and T-cell lymphoma lines has been demonstrated to substantially decrease cytotoxicity caused by CD5-specific CAR T cells, highlighting the importance of the FAS-FASL mechanism [11]. While the perforin and granzyme pathway requires target antigen expression on the cell surface of each tumor cell for its destruction, target antigen expression on each individual cell is not necessary for killing via the FAS-FASL pathway. Once initial antigen encounter has upregulated FASL on a CAR T cell, it can subsequently kill FAS-expressing tumor cells even if they lack the target antigen [1]. Promoting tumor cytotoxicity mediated by FAS-FASL might improve the therapeutic efficacy of CAR T cells directed against tumors with heterogeneous antigen expression and present a way to overcome therapeutic failure due to antigen loss. 

Previous studies have reported that a variety of tumors can downregulate FAS expression on the cell surface in order to escape a T cell-mediated tumor lysis via the FAS-FASL axis [12,13]. FAS expression is known to be positively regulated by the TP53 tumor suppressor [14], whose function is often impaired in tumor cells either by loss-of-function mutations or epigenetic silencing [15]. KDM1A, a histone demethylase originally called LSD1, was the first histone demethylase discovered, and can act as both a transcriptional coactivator and corepressor [16]. Overexpression of KDM1A has been reported in a variety of cancers including neuroblastoma, where the enzyme expression correlates with more aggressive disease [17]. KDM1A has been shown to repress transcriptional activation mediated by TP53 in human cancers [18]. Targeting KDM1A could, therefore, provide a feasible strategy to re-express the TP53-responsive gene, *FAS*, and subsequently increase tumor responsiveness to CAR T cell therapy.

Neuroblastoma is the most common malignant extracranial solid tumor in children, arising from the sympathoadrenal lineage of the neural crest. Disease etiopathology and clinical course are heterogeneous ranging from spontaneous regression to very aggressive tumors that poorly respond to multimodal therapy, classified as high-risk disease. Mortality in children with high-risk disease remains over 50%, and is mainly due to primary or acquired resistance to standard chemotherapy [19]. Treating patients with refractory or relapsed neuroblastoma remains a great challenge in pediatric oncology and new therapeutic approaches are urgently needed. Our group developed CAR T cells targeting the glycosylated CE7 epitope of L1CAM (formerly CD171) [20], which is specifically expressed on tumor cells [20,21]. Children suffering from primary refractory or relapsed neuroblastoma were treated with L1CAM-targeting CAR T cells in an ongoing clinical phase I trial (NCT02311621, https: clinicaltrials.gov, accessed on 27 September 2021).

Here, we investigated whether pharmacologically inhibiting KDM1A can cause TP53-mediated FAS re-expression on neuroblastoma cells to enable antigen-independent killing via the FAS-FASL axis by our L1CAM-specific CAR T cells. Our aim was to epigenetically manipulate neuroblastoma cells to overcome the barrier presented by heterogeneous target antigen expression by unleashing antigen-independent CAR T cell-mediated killing.

## 2. Results

### 2.1. IFNG Release by CAR T Cells Does Not Induce FAS Expression in Neuroblastoma Cell Lines with Intermediate or Low Antigen Expression Levels 

**Figure 1:**
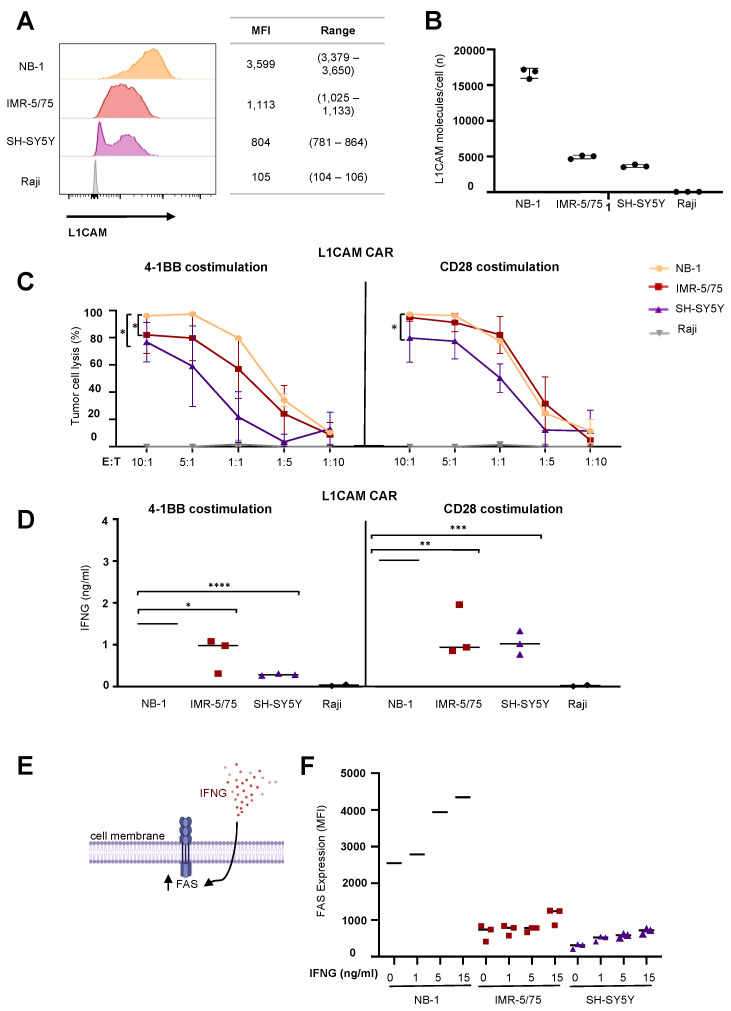
IFNG released by CAR T cells failed to induce FAS expression in neuroblastoma cell lines. (**A**) Target antigen expression of selected neuroblastoma cell lines measured by flow cytometry. (**B**) Target antigen density of selected neuroblastoma cells assessed by standardized QuantiBRITE calibration beads (mean  ±  SD, *n*  =  3). (**C**) CAR T cell-induced tumor cell lysis after 24 h of coculture with neuroblastoma cell lines in different E:T ratios followed by luciferase-based quantification of viable cells (mean  ±  SD, *n*  =  3). (**D**) IFNG release by CAR T cells after 24 h of coculture with neuroblastoma cell lines at an effector:target of 5:1 (mean  ±  SD, *n*  =  3). (**E**) Illustration of IFNG-mediated FAS regulation in tumor cells. (**F**) Flow cytometrically determined FAS expression in neuroblastoma cell lines after IFNG treatment for 24 h (mean  ±  SD, *n*  =  3). * *p*  ≤  0.05, ** *p*  ≤  0.01, *** *p*  ≤  0.001, **** *p*  ≤  0.0001.

To test the efficacy of L1CAM-specific CAR T cells against neuroblastoma cells expressing varying antigen levels, we first quantified L1CAM expression in different neuroblastoma cell lines using flow cytometry and standardized QuantiBRITE calibration beads. We selected cell lines with high (NB-1) or low, and heterogeneous (IMR-5/75, SH-SY5Y) antigen expression (Figure 1A,B) for in vitro assessment of CAR T cell-dependent cytotoxicity and cytokine release. Selected neuroblastoma cell lines were transduced with a GFP-firefly luciferase reporter plasmid to quantify viable tumor cells in a luciferase-based reporter assay. L1CAM-specific second-generation CAR T cells [20] expressing CARs harboring either CD28 (L1CAM-CD28 CAR) or 41BB (L1CAM-4-1BB CAR) as costimulatory domain (Figure S1A) and enriched for homogenous levels of EGFRt expression by cetuximab immunomagnetic positive selection (Figure S1B) were cocultured with the selected neuroblastoma reporter cells in different effector to target (E:T) ratios ranging from 10:1 to 1:10. Cytotoxicity was assessed following 24 h of coculture by measuring the biophotonic signal released by the remaining viable tumor cells. CAR T cell-induced tumor cytotoxicity was generally dependent on target antigen expression level for either CAR construct used, except that CAR T cells equipped with CD28 costimulation achieved equivalent cytotoxicity against cells expressing high or intermediate antigen levels (Figure 1C). CAR T cells equipped with either construct killed none of the L1CAM-negative Raji lymphoma cells serving as negative controls. Cytokine (IFNG) release from CAR T cells after coculture with the three selected neuroblastoma cell lines was quantified by ELISA. IFNG release was proportional to antigen levels expressed on the neuroblastoma cells, independent of the costimulatory domain used (Figure 1D). However, CAR T cells using CD28 costimulation released on average twice as much IFNG than CAR T cells using 4-1BB costimulation. No IFNG was released by CAR T cells exposed to the L1CAM-negative Raji cells, confirming CAR T cell specificity for the L1CAM tumor antigen. Our results demonstrated that low antigen expression impairs CAR T cell efficacy against neuroblastoma cells. 

Exploitation of antigen-independent killing mechanisms by CAR T cells has been described as a way to improve anticancer efficacy against tumors with low antigen expression [1]. IFNG release by CAR T cells sensitizes tumor cells to antigen-independent cytotoxicity by upregulating expression of death receptors such as the FAS receptor (Figure 1E). We tested whether IFNG treatment could upregulate FAS expression on our selected neuroblastoma cell lines. Cultured neuroblastoma cells were exposed to IFNG concentrations ranging from 1 to 15 ng/mL, which was up to 15 times higher than IFNG amounts released by the CAR T cells in coculture experiments. FAS expression on the neuroblastoma cell surface was flow cytometrically assessed after 24 h of IFNG treatment. Interestingly, an IFNG-mediated increase in FAS expression only occurred in NB-1 neuroblastoma cells, which already expressed high FAS levels before IFNG treatment (Figure 1F). The other two neuroblastoma cell lines, with the lower levels of L1CAM target expression, exhibited only very low FAS levels on the cell surface that were only slightly upregulated by IFNG treatment regardless of concentration. These results indicate that low FAS induction on neuroblastoma cells via IFNG hinders exploitation of the antigen-independent FAS-FASL pathway by L1CAM-specific CAR T cells. 

### 2.2. Inhibiting KDM1A Upregulates FAS Expression on Neuroblastoma Cell Lines

**Figure 2:**
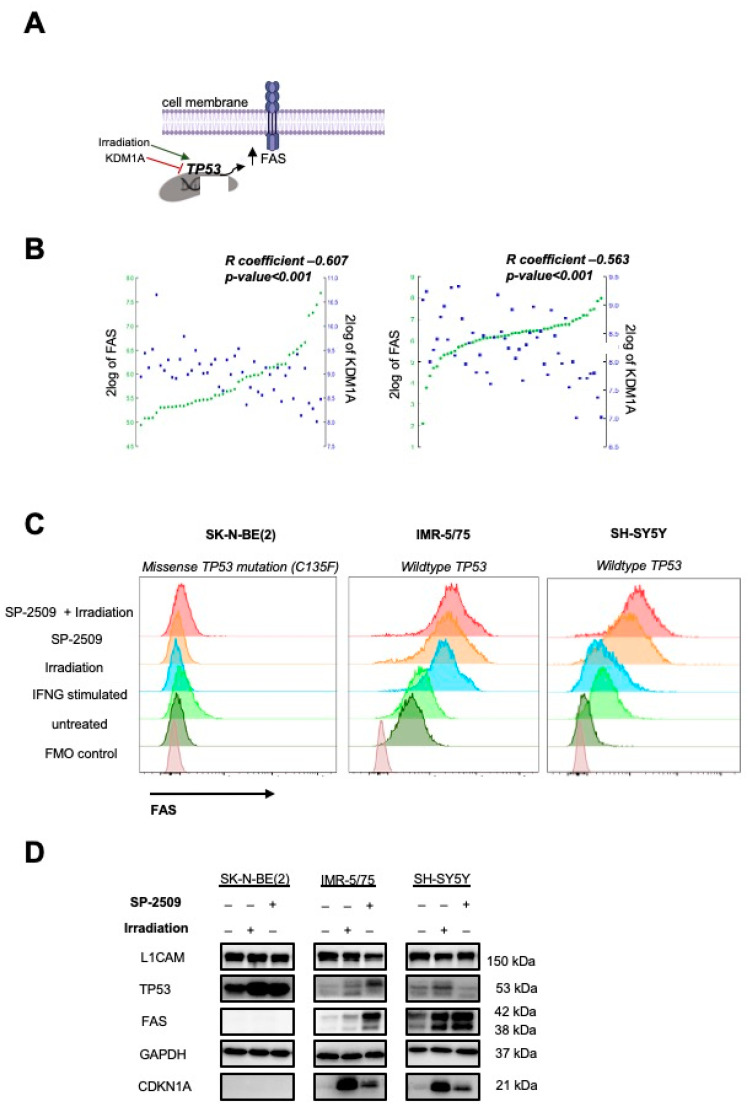
Inhibiting KDM1A strongly enhanced FAS on neuroblastoma cell surface. (**A**) Illustration of FAS regulation in tumor cells. (**B**) Pearson correlations between FAS and KDM1A expression on the left side across 47 samples [22] and on the right side across 51 samples from primary neuroblastomas [23] (source: R2: Genomics Analysis and Visualization Platform (http://r2.amc.nl, accessed on 27 September 2021)). (**C**) Flow cytometrically determined FAS surface expression in neuroblastoma cell lines after 72 h of treatment with 0.5 µM KDM1A inhibitor (SP-2509), irradiation with 2 Gy, or a combination of both (mean  ±  SD, *n*  =  3). (**D**) L1CAM, TP53 and FAS protein expression in neuroblastoma cells after 72 h of treatment with 0.5 µM KDM1A inhibitor (SP-2509), irradiation with 2 Gy, or a combination of both. GAPDH served as loading control.

FAS receptor expression is upregulated by functional TP53 signaling, which is activated after exposure to agents causing DNA double-strand breaks, such as irradiation. KDM1A is an epigenetic regulator capable of suppressing TP53-directed transcriptional activity by demethylating TP53 lysine 370, thus, preventing DNA binding and expression of TP53-responsive genes [18]. We hypothesized that treatment with a small molecule inhibitor of KMD1A should upregulate FAS expression, among other TP53-responsive genes (Figure 2A). Pearson correlation analysis in two independent neuroblastoma cohorts consisting of 47 [22] and 51 [23] tumor samples demonstrated that *KDM1A* and *FAS* expression were negatively correlated (Figure 2B). For subsequent experiments, we used the IMR-5/75 and SH-SY5Y neuroblastoma cell lines because our focus in this study was on tumors with low antigen expression. Since both cell lines harbored wildtype *TP53*, we included the SK-N-BE(2) neuroblastoma cell line, which harbored a missense *TP53* mutation producing a nonfunctional protein, as negative control. All three neuroblastoma cell lines expressed high KDM1A protein levels (Figure S2A). The IC50 of the KDM1A small molecule inhibitor, SP-2509, was assessed in all three cell lines with doses ranging between 0.5 and 500 µM to determine the optimal treatment concentration. As expected, neuroblastoma cell lines harboring functional TP53 were sensitive to lower doses of the inhibitor (1–3 µM), whereas the SK-N-BE(2) cell line with inactive TP53 required higher doses (8.9 µM) to induce cell death (Figure S2B). Treatment with 3 µM SP-2509 upregulated FAS expression in both cell lines with functional TP53 but not the cell line lacking TP53 activity. To investigate whether we could further enhance FAS levels on the tumor cells, we combined the KDM1A inhibitor treatment with low-dose irradiation (2 Gy), but the combination therapy did not induce higher FAS expression levels than KDM1A inhibitor treatment alone (Figure 2C). Low-dose irradiation was also used to induce TP53 activity and FAS surface levels in neuroblastoma cells as a positive control. Western blots for TP53 and FAS showed an increase in TP53 levels in both IMR-5/75 and SK-N-BE(2) cells, but not SH-SY5Y, after SP-2509 treatment. FAS levels were only enhanced by KDM1A inhibitor pretreatment in the cell lines with wildtype *TP53* (IMR-5/75 and SH-SY5Y) (Figure 2D, Figures S3–S5). This finding was consistent with our flow cytometry data and indicates that functional TP53 is required for FAS upregulation via KDM1A inhibition. We concluded that KDM1A inhibition upregulates FAS expression on neuroblastoma cells in a TP53-dependent manner.

### 2.3. Combining KDM1A Inhibition with CAR T Cell Therapy Kills Neuroblastoma Cells Expressing Low Antigen Levels via the FAS-FASL Axis

**Figure 3:**
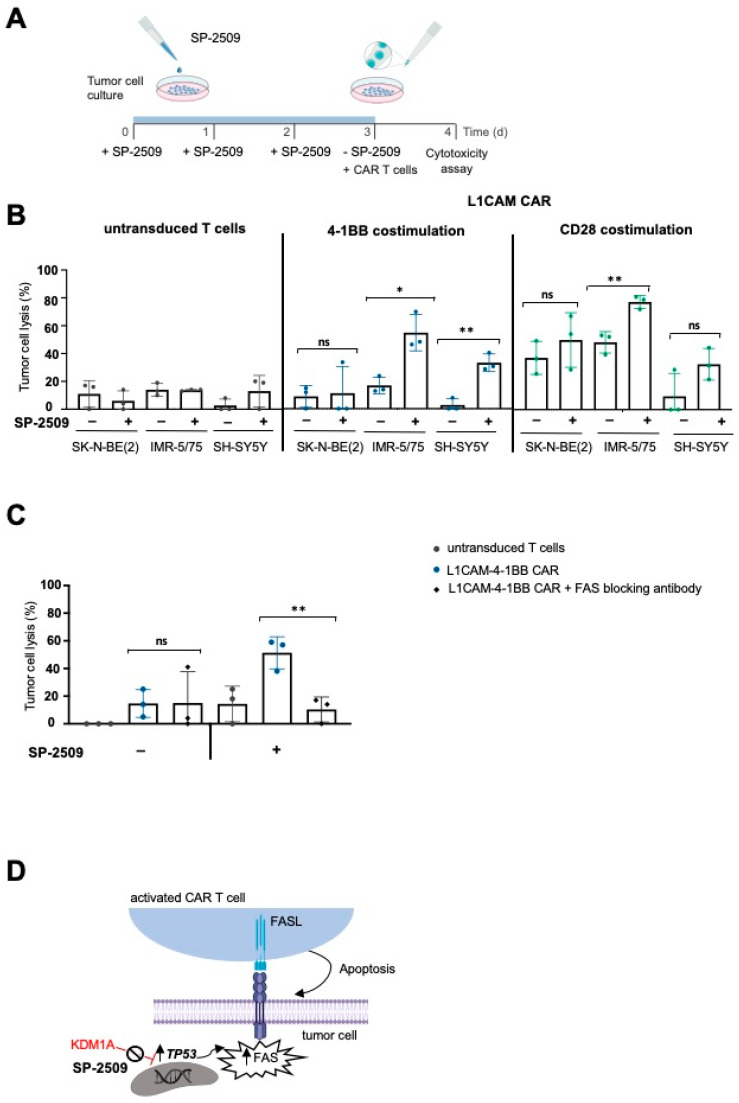
Combining KDM1A inhibition with CAR T cell therapy killed neuroblastoma cells expressing low tumor antigen levels via the FAS-FASL axis. (**A**) Experimental timeline for the combination therapy. (**B**) Killing efficacy of L1CAM-specific CAR T cells after 24 h coculture of neuroblastoma cell lines treated with 0.5 µM SP-2509 (or untreated controls) at an effector:target ratio of 1:5 (mean  ±  SD, *n*  =  3). (**C**) Untreated or KDM1A-pretreated SH-SY5Y cells were incubated for 1 h in the presence or absence of the human neutralizing anti-FAS antibody before measuring the tumor cell lysis by L1CAM-specific CAR T cells cocultured with the tumor cells at an effector:target ratio of 1:5 for 24 h (mean  ±  SD, *n*  =  3). (**D**) Illustration of FAS regulation in tumor cells. ns not significant * *p*  ≤  0.05, ** *p*  ≤  0.01.

To test the functional significance of FAS induction on neuroblastoma cells for CAR T cell efficacy, we combined SP-2509 and L1CAM-specific CAR T cell treatments in vitro. To minimize inhibitor-related cytotoxicity in the experiment, KDM1A inhibitor doses were selected that were high enough to induce FAS expression but low enough to be tolerated by the neuroblastoma cells. FAS expression on IMR-5/75 cells was detected via flow cytometry after treatment with titrated SP-2509 concentrations to select this dose. KDM1A inhibitor doses as low as 0.5 µM could induce FAS expression on tumor cells (Figure S2C), and FAS upregulation by the KDM1A inhibitor remained stable for up to 48 h after inhibitor removal (Figure S2D), allowing subsequent T cell treatment to test combination therapy. To exclude that CAR T cells themselves were affected by KDM1A inhibition, we first assessed KDM1A expression with and without exposure to SP-2509. Untransduced control T cells and CAR T cells expressed much lower KDM1A protein levels than IMR-5/75 neuroblastoma cells (Figure S6A). Although T cells were also sensitive to lower doses of the inhibitor (1.9–2.5 µM) to induce cell death (Figure S6B), 0.5 µM SP-2509 did not impair cytokine release by CAR T cells and was sufficient to upregulate FAS at the tumor site (Figure S6C). For combination experiments, the three neuroblastoma cell lines were exposed to fresh medium or pretreated for 72 h with 0.5 µM SP-2509 before exposure to L1CAM-specific CAR T cells in vitro (Figure 3A). After 24 h of coculture, we measured tumor cell lysis via a bioluminescence-based killing assay. To calculate the impact of FAS upregulation on CAR T cell efficacy, toxicity caused by the inhibitor alone was factored out by using the neuroblastoma cells pretreated for 72 h with SP-2509 alone as the reference baseline. Our results show that cytotoxicity induced by L1CAM-specific CAR T cells was significantly increased by SP-2509 pretreatment of neuroblastoma cells (Figure 3B). We observed a 3.2-fold increase in L1CAM-4-1BB CAR T cell-induced cytotoxicity and a 1.5-fold increase in the L1CAM-CD28 CAR T cell-induced cytotoxicity against neuroblastoma cells expressing intermediate levels of the target antigen (IMR-5/75) compared to CAR T cell treatment alone. The effect was heightened against neuroblastoma cells expressing low L1CAM levels (SH-SY5Y), with a 12.6-fold increase in the L1CAM-4-1BB CAR T cell-directed cytotoxicity and a 3.4-fold increase in L1CAM-CD28 CAR T cell-directed cytotoxicity. Costimulating 4-1BB signaling enhanced the increased cytotoxicity induced by L1CAM-targeting CAR T cells above that from CD28 costimulation. The cytotoxicity induced by either CAR T cell construct against SK-N-BE(2) neuroblastoma cells (lacking functional TP53) was unchanged by SP-2509 pretreatment, supporting that TP53-dependent FAS upregulation is the reason for enhanced CAR T cell efficacy. To exclude that enhanced killing efficacy observed against IMR-5/75 and SH-SY5Y was not due to alterations in tumor antigen expression, L1CAM expression was flow cytometrically assessed 72 h after SP-2509 treatment and shown to be unchanged (Figure S7A,B). To provide further evidence that FAS induction on the tumor cells was the mechanism responsible for enhancing the cytotoxic efficacy of L1CAM-specific CAR T cells, we conducted experiments to specifically block FAS activity. IMR-5/75 neuroblastoma cells, either untreated or pretreated with the KDM1A inhibitor, were incubated with an antibody that blocks FAS binding to its ligand without inducing FAS-directed cell death prior to exposure to CAR T cells. Blockade of FAS activity in the neuroblastoma cells eliminated the increase in tumor cell lysis observed in cells pretreated with the KDM1A inhibitor but did not impact CAR T cell-directed killing of untreated neuroblastoma cells (Figure 3C). Our findings suggested that KDM1A-mediated FAS upregulation at the tumor site is the mechanism behind enhanced CAR T cell efficacy (Figure 3D). 

### 2.4. FAS Upregulation by KDM1A Inhibition Enables CAR T Cells to Eradicate Antigen-Negative Neuroblastoma cells via the FAS-FASL Axis

**Figure 4:**
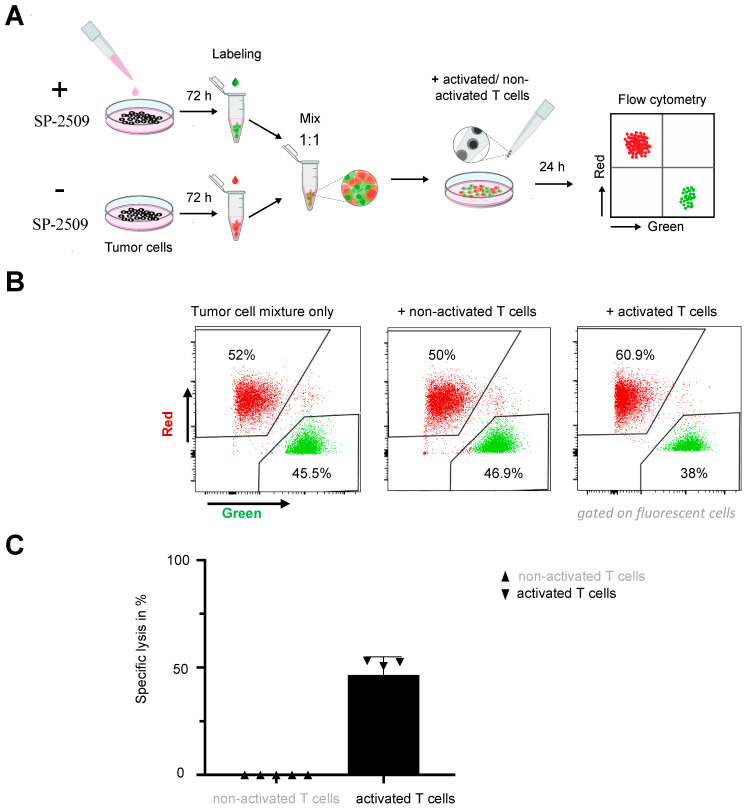
Combination therapy was beneficial for eradicating tumor cells lacking the tumor antigen. (**A**) Experimental timeline for the combination therapy. (**B**) Killing efficacy of activated or unactivated untransduced T cells after 24 h coculture with SH-SY5Y-L1CAM-ko cells treated with 0.5 µM SP-2509 (or untreated controls) at an effector:target ratio of 1:1. (**C**) Specific lysis of SP-2509 pretreated SH-SY5Y-L1CAM-ko cells by unactivated or activated T cells (mean  ±  SD, *n * =  4).

We next explored whether FAS upregulation by KDM1A inhibition was sufficient to kill even antigen-negative neuroblastoma cells via the FAS-FASL axis. To exclude any antigen-related killing, we generated an SH-SY5Y cell model with a complete L1CAM knockout (SH-SY5Y-L1CAM-ko; Figure S8) and exposed the cell model to untransduced T cells stimulated to induce FASL expression (Figure S9). We did not use the L1CAM-negative Raji cell line, derived from a Burkitt lymphoma, for this experiment because the primary focus for our study was to show an effect on a solid tumor such as neuroblastoma. SH-SY5Y-L1CAM-ko cells were pretreated for three days with 0.5 µM SP-2509 and labeled with one fluorescent dye Dil, or alternatively untreated and labeled with a different fluorescent dye DiO, (for flow cytometric identification) before 24 h coculture with either stimulated and unstimulated T cells (E:T = 1:1, Figure 4A). Different ratios (1:1, 5:1 and 10:1) were tested for this experiment because we hypothesized that the previously applied E:T of 1:5 might be unfavorable for the untransduced T cells, which, contrary to CAR T cells, cannot eradicate tumor cells via L1CAM antigen recognition. We showed the results for the experimental design using the lowest number of effector cells (1:1 ratio) that achieved a clear effect. KDM1A-inhibited but not untreated tumor cells were selectively killed by activated T cells, resulting in a ratio shift towards the untreated tumor cells in the resulting population (Figure 4B,C). The ratio of both tumor cell populations remained nearly 1:1 in cultures with unstimulated T cells, validating that FASL-expressing T cells induced cytotoxicity. This finding demonstrated that inhibiting KDM1A was sufficient to assist either endogenous T cells or CAR T cells present in the tumor in killing a heterogeneous tumor cell population containing tumor cells completely lacking expression of the target antigen. Taken together, our results indicated that increasing FAS expression on tumor cells by pharmacologically inhibiting KDM1A activity could improve the efficacy of L1CAM-specific CAR T cell therapy against neuroblastomas with low or heterogeneous levels of target antigen expression by unleashing antigen-independent killing via the FAS-FASL axis.

## 3. Discussion

To date, CAR T cells have shown unprecedented success in the treatment of hematological malignancies [24,25]. However, tumor evasion by downregulation or loss of the target antigen often ultimately leads to therapeutic failure at a later timepoint [26,27]. Therapy success against solid tumors has been very limited, due to antigen heterogeneity among tumor cells, an immunosuppressive tumor microenvironment and limited CAR T cell trafficking and infiltration at the tumor site [28]. Despite all these important factors, the strong heterogeneity in antigen expression within a solid tumor remains one of the greatest challenges limiting CAR T cell efficacy [29,30,31,32]. Developing strategies that extend CAR T cell efficacy against tumor cells with low or absent target antigen expression could provide a route to counteract tumor escape achieved by heterogeneous antigen expression per se or therapy-induced antigen downregulation. Here, we showed that pharmacological KDM1A blockade in neuroblastoma cells with the small molecule inhibitor, SP-2509, dramatically increased FAS cell-surface expression in a strictly TP53-dependent manner. FAS upregulation sensitized neuroblastoma cells to death via the FAS-FASL axis and enabled L1CAM-directed CAR T cells to eradicate antigen-negative tumor cells in vitro.

Several groups have reported failure of CAR T cell therapy against tumors which either exhibited low or heterogeneous antigen expression per se or lost antigen expression after a successful initial treatment. Xu et al. reported, that 10–20% of patients with B-cell acute lymphoblastic leukemia (B-ALL) relapsed after CD19-specific CAR T cell treatment due to emergence of CD19-negative leukemic cells [4]. Similarly, tumor escape via CD22 antigen loss has been observed in up to 30% of patients with B-ALL treated with CD22-specific CAR T cells [33]. Using ALL cell lines with variable CD22 expression, Ramakrishna et al. demonstrated that low CD22 expression (621 molecules/cell) impaired functionality as well as in vivo persistence of CD22-targeting CAR T cells [34]. We used established neuroblastoma cell lines with high, low, or heterogeneous antigen expression, and showed that the in vitro efficacy of L1CAM-directed CAR T cells was significantly reduced when the tumor antigen was less strongly or heterogeneously expressed on tumor cells. This is in line with recent literature reporting reduced CAR T cell activation, proliferation, persistence, and cytokine production as well as reduced cytotoxicity against tumors expressing low levels of target antigen [35]. We also observed differences in the efficacy of our neuroblastoma-specific CAR T cells, depending on the CAR construct employed. The CAR utilizing 4-1BB costimulation performed worse against neuroblastoma cells expressing low antigen levels, compared with the CAR imparting CD28 costimulation. These findings are in line with a report by Majzner et al. that the higher signal strength of CD28-harboring CARs was responsible for their higher efficacy against tumors with low tumor antigen density. They showed that T cells transduced with CD28-harboring CARs manifested more rapid and robust calcium influx than T cells with 4-1BB-harboring CARs [36]. These findings showed that low or heterogeneous tumor antigen expression remains one of the main reasons for therapeutic failure of CAR T cell therapy, especially for the 4-1BB-harboring CAR. To date, novel dual-antigen targeting CARs [37], tandem CARs [38] and CAR T cells secreting a bispecific T cell engager (BiTE) [39] are being investigated to tackle antigen heterogeneity within tumors. However, all these strategies remain dependent on sufficient tumor antigen expression. The advantages of the strategy we propose here, rely on epigenetically reviving an antigen-independent pathway to enhance the CAR T cell potency even in the absence of high-level tumor antigen expression. Since KDM1A is overexpressed on neuroblastoma cells [17], it remains a tumor-specific approach.

The main killing mechanism of CAR T cells is mediated via the perforin and granzyme pathway, which relies on the presence of the target antigen on the tumor cell surface [1]. Blockade of this pathway by the Ca^2+^ chelator, egtazic acid (EGTA), blocks cytotoxic granule exocytosis and almost completely diminishes CAR T cell-mediated tumor cell lysis [11]. The FAS-FASL axis is an alternative antigen-independent killing mechanism, and CAR T cells capable of killing via the FAS-FASL axis have been demonstrated to more efficiently lyse cells from tumors with low or heterogeneous antigen expression [1,40]. This pathway might remain inactive, however, since FAS expression is downregulated on cells in a number of tumors, including gastric [41], colon [13], thyroid [42], and small cell lung [43] carcinomas, consistent with our observations in neuroblastomas. Known mechanisms responsible for FAS absence on the cell surface at the tumor site are transcriptional repression, promoter hypermethylation [44], histone acetylation [45] m and generation of secreted soluble FAS proteins that lack the transmembrane domain via alternative mRNA splicing [46]. Nevertheless, it has been demonstrated that FAS cell surface expression can still be induced in the presence of proinflammatory cytokines. For example, upon antigen recognition, CAR T cells produced proinflammatory cytokines, such as IFNG, which sensitized tumor cells to death receptor-mediated cell death by upregulating FAS expression [47]. Indeed, we observed FAS upregulation in neuroblastoma cells, but only in the presence of very high IFNG concentrations, which would be unrealistic in an in vivo setting. We did not observe FAS upregulation on neuroblastoma cells after exposure to IFNG concentrations equivalent to those produced by T cells upon in vitro tumor cell encounter. An exception was the NB-1 cell line, which expressed high levels of FAS protein on the cell surface even in the absence of IFNG. NB-1 was not included in further experiments due to its high L1CAM surface expression, but we hypothesize that the FAS-FASL axis plays a secondary role in cell lines with high antigen expression. Therefore, we aimed to epigenetically increase FAS levels on neuroblastoma cells to unleash this antigen-independent killing pathway and improve CAR T cell efficacy against neuroblastomas with heterogeneous or low target antigen expression. The epigenetic regulator, KDM1A, is known to suppress TP53-mediated transcriptional activation by maintaining TP53 in an inactive state and preventing its binding to DNA [18]. Müller et al. reported a TP53-responsive element in the *FAS* gene [14]. These reports are in line with our observation, that FAS upregulation via KDM1A inhibition was TP53-dependent. Over 98% of primary neuroblastomas harbor wildtype *TP53* at diagnosis [48]. However, the incidence of dysfunctional TP53 increases at disease relapse. Carr-Wilkinson et al. identified a TP53 dysfunction in almost half the cases (49%) of relapsed neuroblastoma in 41 patients, with the dysfunction stemming from a TP53 mutation in 15% of the cases and an upstream defect (affecting p14ARF or MDM2) in 35% of the cases [49]. TP53 activation via pharmacological MDM2 inhibition was recently shown to sensitize tumors to T cell-mediated killing [50]. Other agents suitable to revive TP53 functionality might, therefore, present alternative strategies to improve CAR T cell therapeutic efficacy by promoting antigen-independent killing via the FAS-FASL axis.

Irradiation is also known to upregulate FAS expression by inducing TP53 protein activity [51]. One study in nasopharyngeal carcinoma shows that irradiation over time increases epigenetic silencing of *TP53* via the DNMT3B DNA methyltransferase [52]. Radiation therapy not only induces a number of early side effects due to damage of surrounding healthy tissue but can also cause late adverse effects such as second malignancies. We showed that KDM1A inhibition in IMR-5/75 neuroblastoma cells induced FAS cell surface expression to higher levels than irradiation in a direct comparison. Maecker et al. demonstrated that epigenetic silencing of TP53 gene targets, such as *FAS*, can occur via TP53-independent mechanisms in cells that retain functional TP53 [53]. Huang et al. reported decreased histone acetylation and increased H3K9Me3 at the *FAS* promoter in patients with idiopathic pulmonary fibrosis, where treatment with histone deacetylase (HDAC) inhibitors restored FAS expression [45]. These findings from us and others demonstrate the critical role histone modification plays in epigenetically modulating *FAS* expression. 

A possible limitation of the strategy we propose here is the biological role that KDM1A has in normal hematopoietic and neuronal stem cells [14]. Inhibiting KDM1A in these normal tissues could potentially cause treatment toxicity. However, the KDM1A inhibitor, tranylcypromine (TCP), is in clinical use as a monoamine oxidase (MAO) inhibitor to treat therapy-resistant depression. TCP was later identified as an irreversible and weak KDM1A inhibitor [54]. In recent clinical trials, treatment with 40 mg of TCP was well tolerated with an acceptable safety profile in patients with relapsed/refractory AML and myelodysplastic syndromes [55]. We selected the SP-2509 inhibitor, which allosterically inhibits KDM1A by targeting its H3 pocket, for this study because of its high specificity for KDM1A. Furthermore, tumor-bearing mice treated with SP-2509 (15–25 mg/kg b.i.w. via IP injection) for three weeks exhibited no signs of toxicity [56], suggesting an acceptable safety profile. 

Combining the inhibitor, SP-2509, with neuroblastoma-specific CAR T cells improved their efficacy, especially against neuroblastoma cells with low levels of tumor antigen, where insufficient upregulation of FAS by T cell-produced proinflammatory cytokines was observed. Ectopic FAS expression at the tumor site was recently shown to improve CAR T cell efficacy against embryonal carcinomas by enabling antigen-independent FASL-dependent tumor cell lysis [40]. Evidence for FAS-dependent bystander killing of antigen-negative tumors by T cells was also delivered by Upadhyay et al., who postulated that combining CAR T cells with small molecules targeting the FAS pathway may potentiate this mechanism [57]. Monoclonal antibodies that act as a FAS agonist have also been developed as an alternative strategy to activate tumor cell killing via the FAS-FASL axis. However, systemic toxicities caused by FAS expression in normal tissues and a 100-fold lower antibody efficacy compared to FASL expressed on T cells limited the further development of this approach [58]. In contrast, the FASL-mediated killing mechanism exploited by CAR T cells is strictly regulated, because activation-dependent upregulation of FASL on CAR T cells can only occur at the tumor site upon antigen encounter [1]. Overall, we propose that a combination of small molecule KDM1A inhibitors and CAR T cell therapy might present a promising strategy to efficiently attack neuroblastoma and other solid tumor entities with low or heterogeneous antigen expression by enabling eradication of an antigen-negative tumor fraction. Investigation of the toxicity and effectiveness of this strategy in vivo will be necessary before translating these findings to clinical therapy.

## 4. Materials and Methods 

### 4.1. Generation of L1CAM-Specific CAR T Cells

CAR lentiviruses were propagated in 293T cells as described before [59] using the previously generated L1CAM-specific CE7-CAR cloned into the SIN epHIV7 lentiviral vector plasmid [20]. The single-chain variable fragment was codon optimized and subsequently linked to a 12-amino acid (short) spacer domain from the human IgG4 hinge. The spacer domain connected the antigen-binding domain to CD28 transmembrane domain followed by the signaling module containing either the 4-1BB or CD28 endodomain and the CD3zeta cytoplasmic domain. CAR constructs were linked downstream to a T2A self-cleaving peptide and truncated epidermal growth factor receptor (EGFRt) to allow CAR T cell detection and enrichment. CAR T cells were generated using T cells from healthy donors (Charité ethics committee approval EA2/262/20), then cryopreserved and expanded as previously described [60]. Untransduced T cells were used as negative controls alongside CAR T cells in experiments. Functional in vitro assays were conducted between 11 and 16 days after initiating T cell expansion.

### 4.2. Neuroblastoma Cell Lines 

The human NB-1, IMR-5/75 and SH-SY5Y neuroblastoma cell lines were purchased from the *Deutsche Sammlung von Mikroorganismen und Zellkulturen* (Braunschweig, Germany) and cultivated in Roswell Park Memorial Institute (RPMI) 1640 media (Gibco Thermo Fischer Scientific, Waltham, MA, USA) supplemented with 10% fetal bovine serum (MilliporeSigma, Burlington, MA, USA) and 500 µg/mL geneticin (G418, MilliporeSigma, Burlington, MA, USA). The human SK-N-BE(2) neuroblastoma cell line was purchased from the American Type Culture Collection (Manassas, VA, USA), and cultivated in Dulbecco’s Modified Eagle Medium (DMEM, Thermo Fischer Scientific, Waltham, MA, USA) supplemented with 10% fetal bovine serum (MilliporeSigma, Burlington, MA, USA) and 500 µg/mL G418 (MilliporeSigma, Burlington, MA, USA). Cell line identity was confirmed using short tandem repeat DNA genotyping (Eurofins Scientific SE, Luxemburg, Luxemburg), and cultures were routinely tested for mycoplasma contamination using the PlasmoTest Kit (Thermo Fischer Scientific, Waltham, MA USA). All cells were cultured in a humidified atmosphere containing 5% CO_2_ at 37 °C.

### 4.3. CRISPR-Mediated Generation of L1CAM Knockout in SH-SY5Y Neuroblastoma Cells

Three high-ranking guide RNAs targeting L1CAM exon 1 were designed using Dr. Feng Zhang’s online CRISPR design tool [18] and synthesized with BbsI restriction overhangs. Guide RNA sequences excluding the BbsI-compatible overhangs are: RNA1, GACTGTTCCGTGATGACAGG and RNA2, CACCGCCTCGGGGATCTGGATAAGC. Respective oligonucleotides were annealed and ligated into the BbsI-digested pSpCas9(BB)-2A-Puro (PX459) V2.0. Generated vectors were validated by sequencing before transfection into SH-SY5Y neuroblastoma cells using the Effectene transfection reagent kit (cat #301425, Qiagen, Venlo, Netherlands) according to the manufacturer’s instructions. Briefly, 5 × 10^6^ SH-SY5Y neuroblastoma cells were seeded per well into a 6-well plate 24 h before transfection using 100 µL Effectene buffer, 1 μg plasmid DNA, 2 μL Enhancer, and 7.5 μL Effectene per well. Puromycin selection (0.5 μg puromycin/mL complete medium) was conducted 3 to 6 days after transfection. Single-cell clones were established from FACS-sorted L1CAM-negative cells. One single-cell clone with a bi-allelic L1CAM knockout confirmed by sequencing was selected and propagated for experiments.

### 4.4. IFNG Stimulation

Tumor cells were seeded into 6-well plates for 24 h treatment in triplicate with 15 ng/mL IFNG (#300-02-250UG, PEPROTECH, Hamburg, Germany). 

### 4.5. KDM1A Inhibition

Tumor cells were seeded into 10 or 15 cm dishes and treated with 0.5–3.0 µM KDM1A inhibitor SP-2509 (M60160-2s Xcessbio, Chicago, IL, USA) for 3 days, during which treatment medium was replenished for cells every 24 h. Irradiated cells were used as a positive control for the induction of FAS. The cells were exposed to a total dose of 2 Gy at a rate of 0.848 Gy/min using a gamma irradiator (GSR D1; GSM GmbH) operating with a maximum surface dose rate of <5 μSv/h at a maximum activity of 200TBq Cs-137.

### 4.6. FAS Blockade

For antibody-based FAS-FASL blockade, 5 × 10^5^ untreated or KDM1A-inhibited tumor cells were seeded into 24-well plates in triplicate and incubated with the anti-FAS antibody (CAT# 05-338, clone ZB4, MERCK, Darmstadt, Germany) at a concentration of 500 ng/mL for 1 h prior to coculture with CAR T cells. 

### 4.7. FASL Induction in Untransduced T Cells

Untransduced T cells (3 × 10^7^) were stimulated for 1 h with 20 ng/mL phorbol-12-myristate-13-acetate (#P8139 MilliporeSigma, Burlington, MA, USA) and 1 µg/mL ionomycin (#I0634 MilliporeSigma, Burlington, MA, USA), then for further 3 h with 10 µg/mL brefeldin A (#347688, BioLegend, San Diego, CA, USA) and 5 µg/mL monensin (#M5273 MilliporeSigma, Burlington, MA, USA) in culture medium at 37 °C with 5% CO_2_. 

### 4.8. Western Blotting

Tumor cells were detached by trypsin, washed twice with PBS, and lysed in RIPA buffer containing 15 mM HEPES, 150 mM NaCl, 10 mM EGTA and 2% Triton-X100 and protease inhibitors (#11697498001, MilliporeSigma, Burlington, MA, USA). Proteins (20 μg total protein mixed with SDS sample buffer) were resolved on SDS–PAGE and transferred onto nitrocellulose membranes. Blots were blocked with 5% non-fat dry milk diluted in Tris-buffered saline (pH 7.4) with 0.05% Tween 20 for 1 h at room temperature before incubating overnight at 4 °C with primary antibodies against L1CAM (diluted 1:1000, mouse UJ127.11, Thermo Fischer Scientific, Waltham, MA USA), KDM1A (diluted 1:1000, rabbit #2139, Cell Signaling Technology, Danvers, MA, USA), FAS (diluted 1:500, rabbit ab82419, abcam, Cambridge, UK), TP53 (diluted 1:1000, mouse sc-126, Santa Cruz Biotechnology, Dallas, TX, USA) and GAPDH (diluted 1:5000, mouse sc-32233, Santa Cruz Biotechnology, Dallas, TX, USA). Blots were incubated with horseradish peroxidase-conjugated secondary antibodies (diluted 1:5000, anti-mouse: #730020, Thermo Fischer Scientific, Waltham, MA USA, anti-rabbit: #W10809, Thermo Fischer Scientific, Waltham, MA USA) for 2 h at room temperature, then developed using the ECL kit (RPN2232 GE Healthcare, Chicago, IL, USA). Signal intensities were measured using the Fusion FX software (Vilber Lourmat, Eberhardzell, Germany).

### 4.9. Cell Viability Assay 

For determining the half-maximal inhibitory concentration (IC50) of SP-2509, a total of 10,000–25,000 tumor cells were seeded into flat-bottomed 96-well plates in triplicate. Cells were allowed to settle for 3 h before inhibitor (1:10 and 1:100 dilutions, range from 1 to 300 µM) was added for 72 h treatment. Untreated cells were used as a negative control. The CellTiter-Glo^®^ Luminescent Cell Viability Assay (G9242 Promega, Madison, WI, USA) was performed according to manufacturer’s instructions. Viability was calculated relative to untreated tumor cells using GraphPad prism (Version 6.00, https://www.graphpad.com/scientific-software/prism/, accessed on 27 September 2021). 

### 4.10. Flow Cytometry 

Cell-surface expression of FAS and FASL was detected by the respective fluorophore-conjugated monoclonal antibody (#305611, DX2 and #306407, NOK-2; BioLegend, San Diego, CA, USA). L1CAM cell-surface expression was detected by the fluorophore-conjugated monoclonal antibody (#130-100-691, REA163, Miltenyi Biotec, Bergisch Gladbach, Germany) and standardized QuantiBRITE calibration beads (#655050, BioLegend, San Diego, CA, USA). Activation was assessed by fluorophore-conjugated monoclonal antibodies detecting CD8 (#344741, SK1, BioLegend, San Diego, CA, USA) and CD137 (#309819, 4B4-1, BioLegend). Flow cytometry was performed on an LSRFortessa X-20 (BioLegend, San Diego, CA, USA) and data were processed using FlowJo software (Tree Star Inc., Ashland, OR, USA). Samples were acquired with at least 10,000 alive events. Dead cells were excluded from analyses using the LIVE/DEAD^TM^ Fixable Green Dead Cell Stain Kit (cat#L23101, Thermo Fischer Scientific, Waltham, MA, USA). 

### 4.11. Flow Cytometry-Based Cytotoxicity Assay 

Following KDM1A inhibitor pretreatment (or control condition without treatment), SH-SY5Y tumor cells were labeled with Vybrant™ Dil and DiO Cell-Labeling Solution (Thermo Fischer Scientific, Waltham, MA, USA) according to the manufacturer’s instructions, mixed in a 50:50 ratio with each other, and seeded at 5 × 10^5^ tumor cells/well of a 12-well plate. Stimulated untransduced T cells were added (E:T ratio of 1:1) after tumor cells had settled 3 h. The ratio of living KDM1A-treated to untreated tumor cells remaining in each well was flow cytometrically determined after 24 h. Specific lysis was calculated using the formula, [1 − (Ratio untreated:treated tumor cells cocultured with unstimulated T cells/Ratio untreated:treated tumor cells cocultured with stimulated T cells)] × 100.

### 4.12. Functional Assays

For cytokine release assays, 2 × 10^5^ T cells were seeded together with tumor cells at a 1:5 E:T ratio. Media conditioned by cocultures were collected after 24 h and stored at −80 °C until analysis of IL2 and IFNG using the OptEIA™ ELISA (BioLegend, San Diego, CA, USA) according to manufacturer’s instructions. CAR T cell-induced cytotoxicity was quantified in a biophotonic luciferase assay in which the neuroblastoma cells stably transduced with a GFP-ffLuc_epHIV7 reporter, served as tumor target cells. Target cells were pretreated 72 h with 0.5–3 µM KDM1A inhibitor (technical triplicates), then cocultured with untransduced T cells (controls) or CAR T cells. After 24 h, 0.14 mg D-luciferin (PerkinElmer Inc., Waltham, MA, USA) was added, and tumor cell lysis was determined as described before [60].

### 4.13. Statistical Analyses

Differences in cytotoxic activity, cell surface marker expression, and cytokine release between treatment and control groups were analyzed using paired or unpaired Student’s T test in GraphPad prism 8 software (GraphPad Software, La Jolla, CA, USA). *p* values < 0.05 were considered statistically significant. All experiments were independently repeated (*n* = 3).

## 5. Conclusions

Epigenetic manipulation via KDM1A blockade upregulates FAS receptor expression in neuroblastoma cells with functional TP53 signaling. FAS upregulation sensitized neuroblastoma cells to FAS-FASL-dependent death, and enabled L1CAM-directed CAR T cells to eradicate antigen-negative tumor cells in vitro. This strategy demonstrates the potential to increase CAR T cell efficacy against high-risk neuroblastoma and potentially other solid tumors known to overexpress KDM1A.

## Data Availability

The data presented in this study are available on request from the corresponding author.

## Supplementary Materials

The following are available online at https://www.mdpi.com/article/10.3390/cancers13215489/s1, Figure S1: CAR constructs and transduction efficacy, Figure S2: Drug-mediated KDM1A inhibition induces FAS expression in neuroblastoma cell lines, Figure S3: Drug-mediated KDM1A inhibition induces the FAS protein expression in neuroblastoma cell lines. Figure S4: Drug-mediated KDM1A inhibition induces TP53 and CDKN1A protein expression in neuroblastoma cell lines. Figure S5: L1CAM protein expression remains the same after drug-mediated KDM1A inhibition in neuroblastoma cell lines. Figure S6: KDM1A inhibitor does not impair CAR T cell effector function, Figure S7: KDM1A inhibition does not affect target antigen expression on neuroblastoma cells, Figure S8: Validation of L1CAM knockout in SH-SY5Y cell model, Figure S9: FASL expression is upregulated on activated primary T cells.
