# Supplementary material for: Inhibiting Lysine Demethylase 1A Improves L1CAM-Specific CAR T Cell Therapy by Unleashing Antigen-Independent Killing via the FAS-FASL Axis
Source: Cancers (Basel). 2021 Oct 31;13(21):5489. doi: 10.3390/cancers13215489 (PMC8583435; doi:10.3390/cancers13215489)
Supplement: Supplementary file 1 [file cancers-13-05489-s001.zip › cancers-1418665-supplementary.pdf]

SUPPLEMENTARY MATERIALS Sulejmani et al.

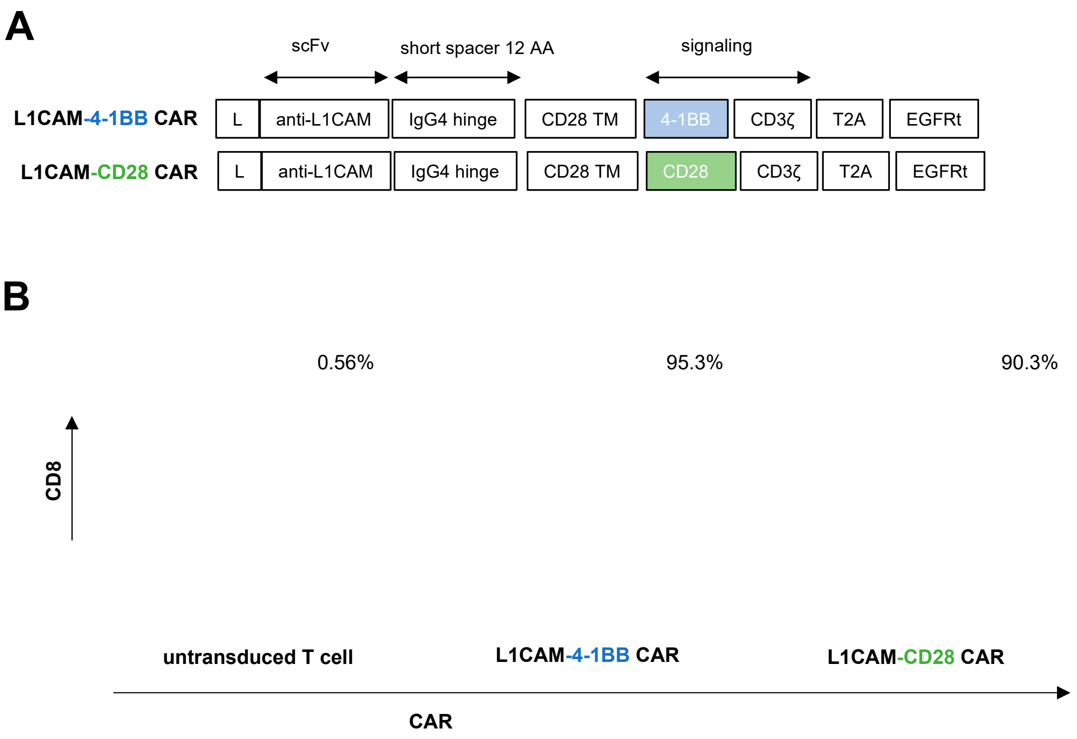

Supplementary Figure S1: ***CAR constructs and transduction efficacy.*** **A.** Schematic illustration of CAR T cell constructs. **B.** Transduction efficiency for CAR constructs and negative control was assessed by flow cytometry (mean ± SD, n = 3).

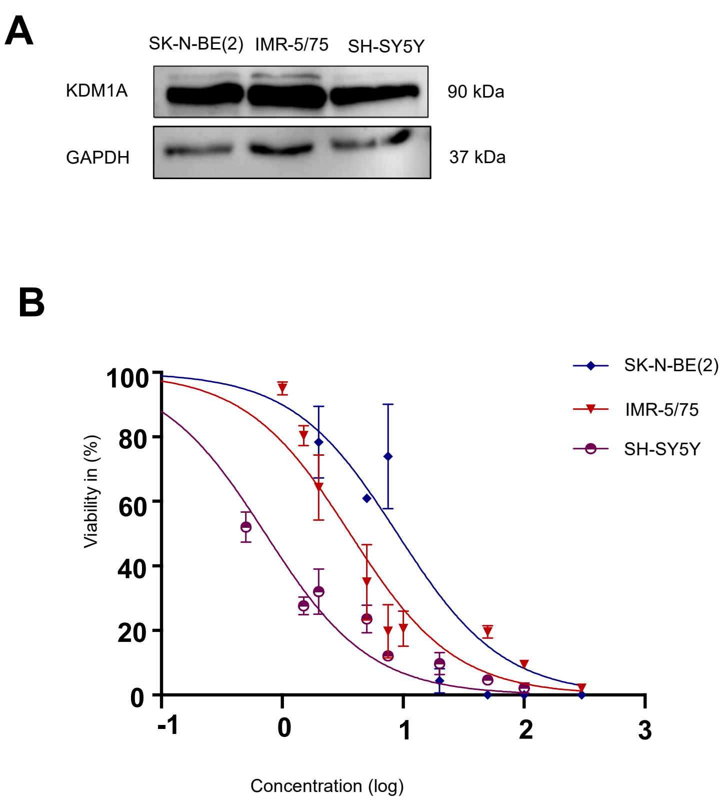

Cell line	SK-N-BE(2)	IMR-5/75	SH-SY5Y
IC50 value (µM)	8.9	3.6	0.7

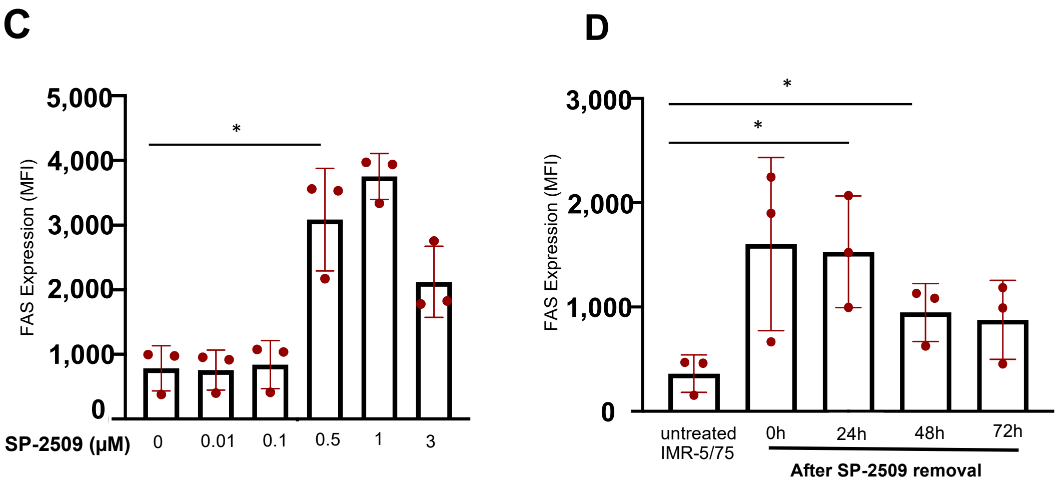

Supplementary Figure S2: ***Drug-mediated KDM1A inhibition induces FAS expression in neuroblastoma cell lines.*** **A.** Western blots showing KDM1A expression in the selected neuroblastoma cell lines. GAPDH was used as a loading control. **B.** Dose-response curves and calculated IC50 scores for the selected neuroblastoma cell lines treated 72 h with the KDM1A inhibitor, SP-2509. **C.** Flow cytometric determination of FAS expression on the surface of IMR-5/75 cells after 72 h treatment with the indicated concentrations of the SP-2509 inhibitor. MFI=mean fluorescence intensity **D.** Flow

cytometric determination of FAS expression on the surface of IMR-5/75 cells after 0, 24, 48 and 72 h removal of the inhibitor by replacing medium after washing cells once (bars show mean ± SD, n = 3). *$p \leq 0.05$.

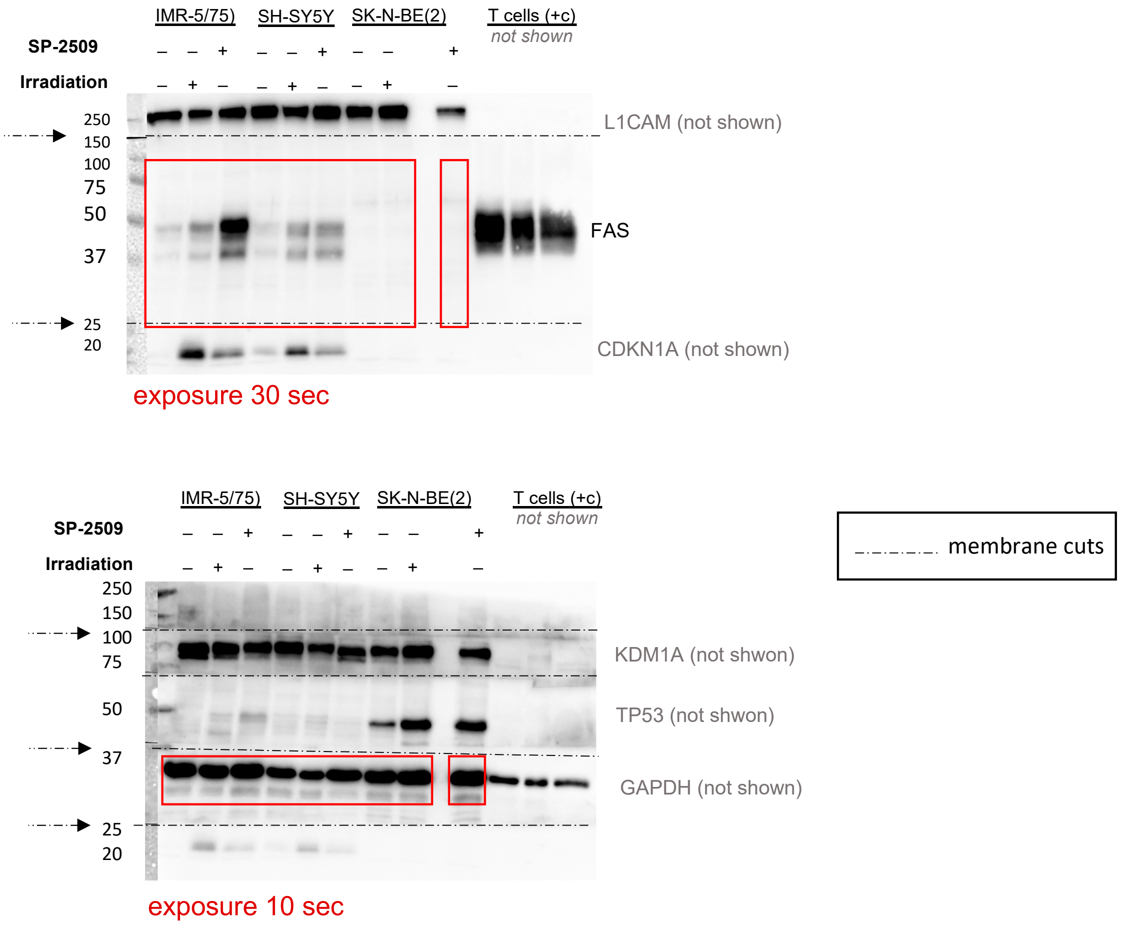

Supplementary Figure S3: ***Drug-mediated KDM1A inhibition induces the FAS protein expression in neuroblastoma cell lines.*** After protein transfer, membrane was cut as indicated. Individual membrane pieces were stained with L1CAM, FAS and CDKN1A antibody as indicated. After development, membrane pieces were stripped, the middle membrane piece was cut as indicated and individual membrane pieces were incubated with either FAS, KDM1A, TP53 and GAPDH antibody as indicated. Since there is a skipped lane between sample SK-N-BE(2) irradiated and SK-N-BE(2) treated with SP-2509, the empty lane was removed in the paper figure. The corresponding loading control (GAPDH) can be seen in the lower picture. The T cells were used as a positive control regarding FAS protein expression.

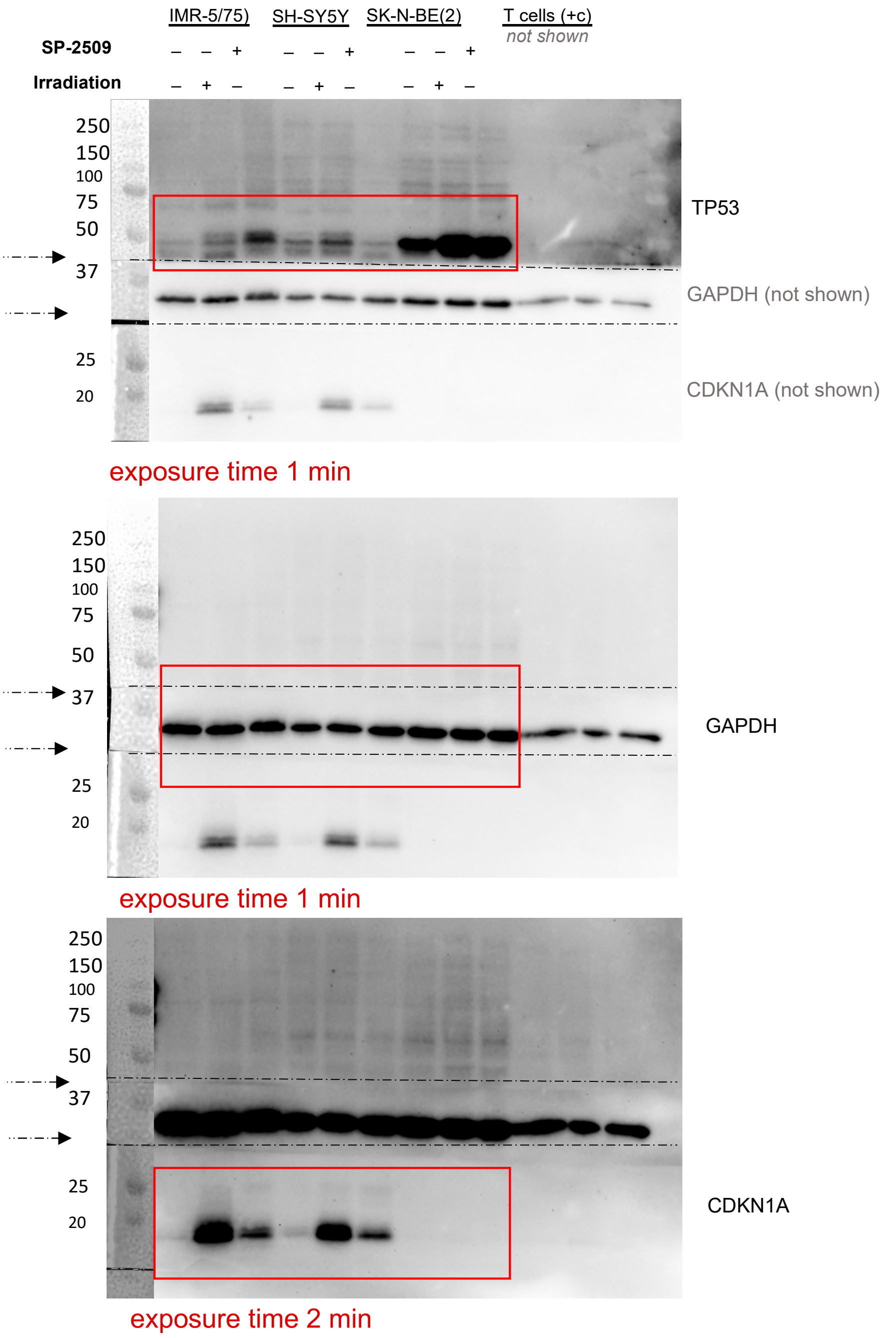

Supplementary Figure S4: ***Drug-mediated KDM1A inhibition induces TP53 and CDKN1A protein expression in neuroblastoma cell lines.*** After protein transfer, membrane was cut as indicated. Individual membrane pieces were stained with TP53, GAPDH, and CDKN1A antibody as indicated.

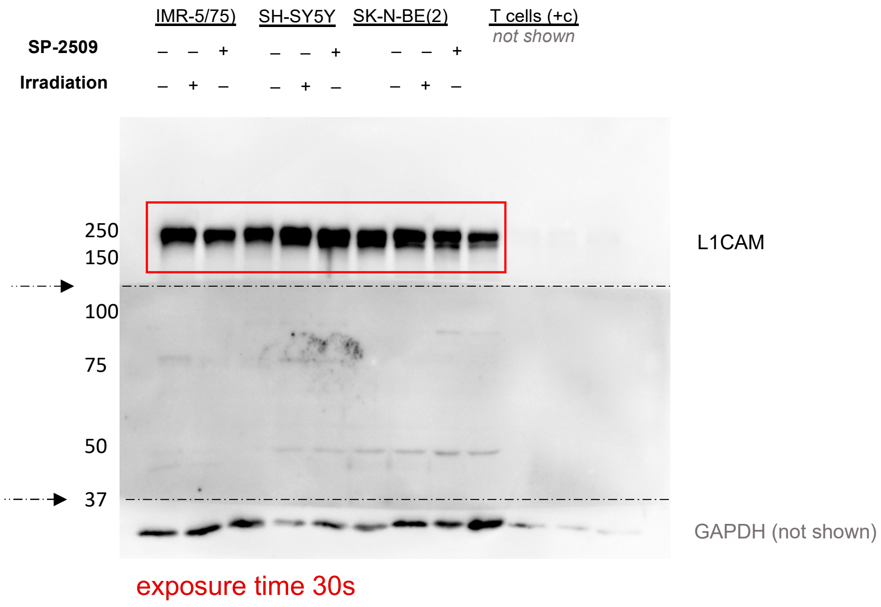

Supplementary Figure S5: *L1CAM protein expression remains the same after drug-mediated KDM1A inhibition in neuroblastoma cell lines.* After protein transfer, membrane was cut as indicated. Individual membrane pieces were stained with L1CAM and GAPDH antibody as indicated.

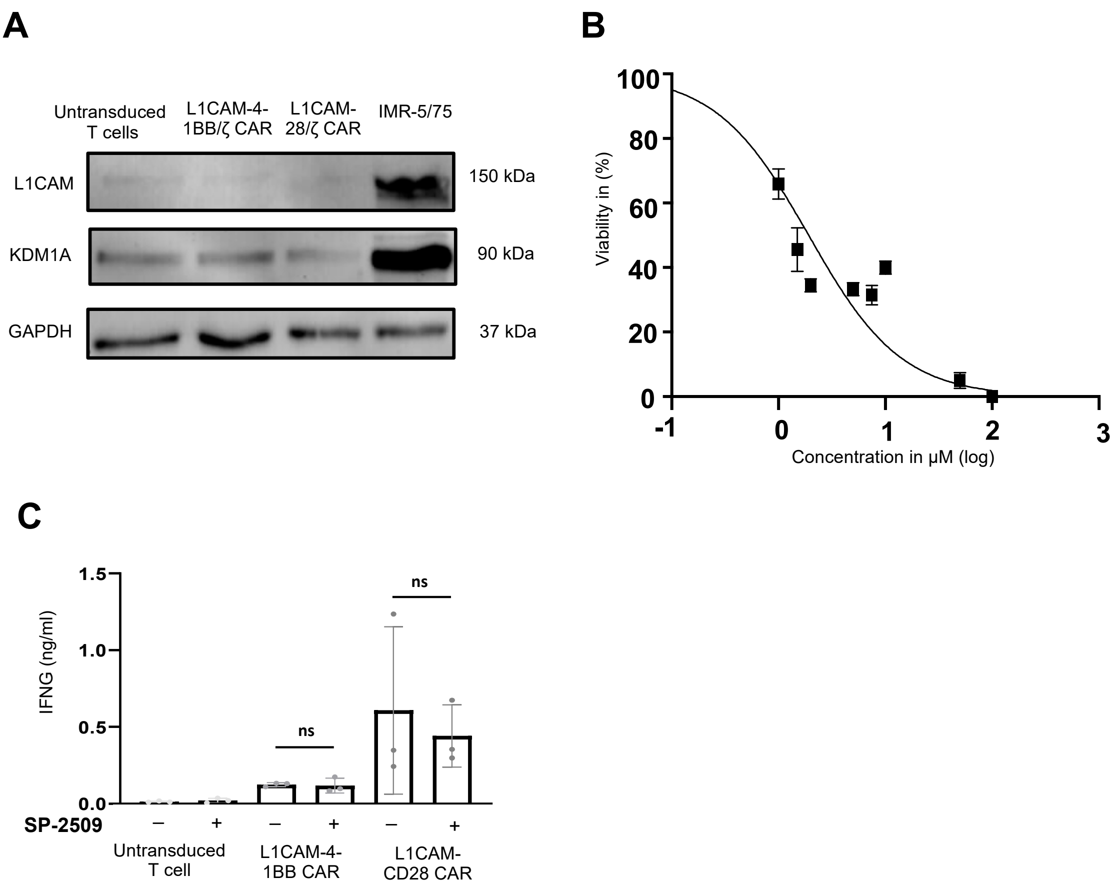

Supplementary Figure S6: ***KDM1A inhibitor does not impair CAR T cell effector function.*** **A.** KDM1A and L1CAM protein expression was determined by western blotting whole-cell extracts from primary T cells and indicated cell lines. GAPDH was used as a loading control. **B**. Dose-response curve indicating calculated IC50 of primary T cells treated 72 h with the KDM1A inhibitor, SP-2509. **C.** IFNG release from untreated and KDM1A pre-treated CAR T cells after 24 h of coculture with tumor cells assessed by ELISA. Bars show mean ± SD from 3 biological replicates. $^*p \leq 0.05$.

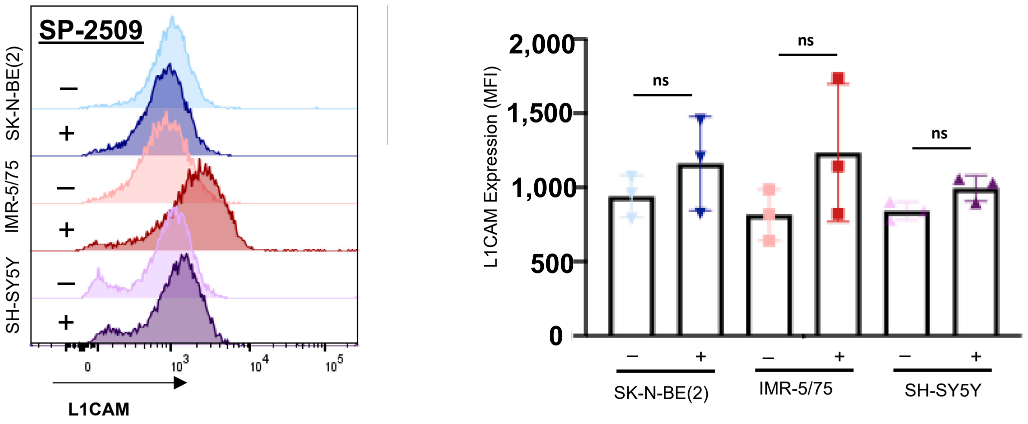

Supplementary Figure S7: ***KDM1A inhibition does not affect target antigen expression on neuroblastoma cells.*** Cell surface L1CAM expression on neuroblastoma cell lines used in this study was flow cytometrically determined 72 h after treatment with the KDM1A inhibitor. A representative histogram plot is shown for each cell line. Bar diagram summarizes mean fluorescence intensity (MFI) values acquired in three individual experiments (mean ± SD).

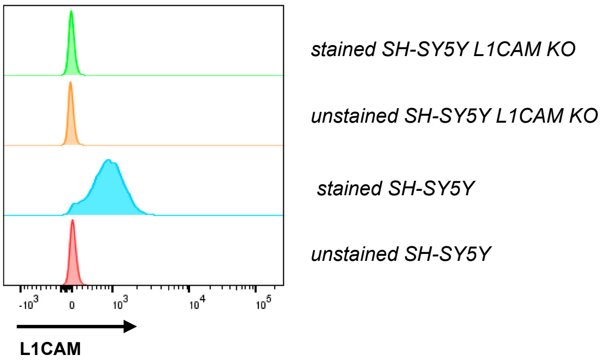

Supplementary Figure S8: ***Validation of L1CAM knockout in SH-SY5Y cell model.*** Flow cytometric assessment of L1CAM surface expression after CRISPR-mediated L1CAM knockout in the SH-SY5Y neuroblastoma cell model**.** Shown are histogram plots of the parental SH-SY5Y neuroblastoma cell line and the knockout clone, either stained with L1CAM fluorophore-conjugated monoclonal antibody or unstained (mean ± SD, n = 3).

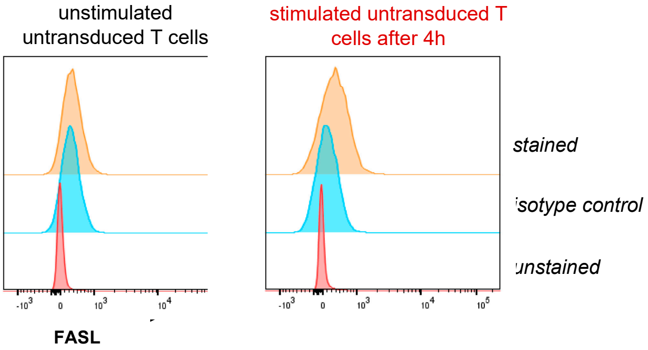

Supplementary Figure S9: ***FASL expression is upregulated on activated primary T cells.*** Primary T cells were either stimulated with PMA/ionomycin for 4 h or were left untreated and subsequently stained with FASL fluorophore-conjugated monoclonal antibody to assess the FASL surface expression. Unstained T cells served as a control. (mean ± SD, n = 3).
